# How patients with severe mental illness experience care provided by psychiatric mental health nurse practitioners

**DOI:** 10.1097/JXX.0000000000000867

**Published:** 2023-04-27

**Authors:** Loes van Dusseldorp, Marieke Groot, Anneke van Vught, Peter Goossens, Hugo Hulshof, Jeroen Peters

**Affiliations:** 1 Expertise Center for Pain and Palliative Medicine, Radboud University Medical Centre, Nijmegen, The Netherlands; 2 Master Advanced Nursing Practice, HAN University of Applied Science, Nijmegen, The Netherlands; 3 HR University of Applied Science, Rotterdam, The Netherlands; 4 HAN University of Applied Science, Nijmegen, The Netherlands; 5 Dimence Group Mental Health Care, Deventer, The Netherlands; 6 University of Gent, Ghent, Belgium

**Keywords:** Metaphors, nurse practitioner, phenomenological perspective, severe mental illness

## Abstract

**Background:**

Previous studies in somatic health care revealed that patients find nurse practitioners reliable, helpful, and empathic and feel empowered, at peace, and in control when cared for by nurse practitioners (NPs). Only one study so far considered what value people with severe mental illness (SMI) attached to treatment by a psychiatric mental health nurse practitioner (PMHNP).

**Purpose:**

To explore what meaning people with SMI associate with the care provided by a PMHNP.

**Methodology:**

A qualitative study from a phenomenological perspective was conducted, in which 32 people with SMI were interviewed. Data were analyzed using Colaizzi's seven-step method and the metaphor identification procedure (MIP).

**Results:**

Eight fundamental themes emerged: (1) impact of the PMHNP on well-being, (2) feeling connected with, and (3) acknowledged by the PMHNP; (4) the PMHNP's care (not) needed; (5) perception of the PMHNP as a person; (6) shared decision-making; (7) PMHNP's expertise; and (8) flexibility of contact with the PMHNP. MIP analysis revealed six metaphors: PMHNP is a travel aid, means trust, is a combat unit, means hope, is an exhaust valve, and a helpdesk/encyclopedia.

**Conclusions:**

The interviewees highly appreciated the treatment and support by the PMHNP for the impact on their well-being. Thanks to the connection with and recognition by the PMHNP, they felt empowered, human, and understood. Challenged by the PMHNP, they focused on possibilities to strengthen self-confidence and self-acceptance.

**Implications:**

For further positioning of and education for PMHNPs, it is recommended to consider the meaning people with SMI associate with treatment and support by a PMHNP.

## Introduction

### Background

A psychiatric mental health nurse practitioner (PMHNP) acts at the intersection of care and cure. The Canadian Medical Education Directions for Specialists (CanMEDS), on which the curriculum the Netherlands is based, distinguishes seven core competencies a nurse practitioner should possess such as Clinical Expertise, Communication, and Health Advocacy (Kappert & de Hoop, 2019). In daily practice, nurse practitioners in general fulfill a broad range of roles—depending on the work setting, their clinical discipline, collaboration with the health care team, and the organizational policy regarding task allocation and professional development (ter Maten-Speksnijder et al., 2014). Since 2017, PMHNPs in the Netherlands act as coordinating practitioners to improve quality of psychiatric mental health care (Boeijen et al., 2021). The National Health Care Institute (2020) defines a coordinating practitioner as the first point of contact for patients who are responsible for the mainstay of the treatment and the coordination with other treating practitioners. In case of multidisciplinary treatment, a coordinating practitioner also provides a substantial part of the treatment, e.g., medication management or psychotherapy (Kappert & de Hoop, 2019).

In the Netherlands, 1.7% of the population suffer from severe mental illness (SMI; Delespaul & de Consensusgroep, 2013). Approximately 75% of them (210,700 people) receive—with different frequencies and duration of contact—either inpatient or outpatient specialized medical psychiatric treatment. For example, (Flexible) Assertive Community Treatment, Active Recovery Triad provided by long-term care mental health care institutions, professionally supported and secure small-scale housing projects, or ambulatory care while living independently (Akwa, 2021).

Apart from specialized medical psychiatric treatment, many of them need support and guidance regarding social relationships, work, housing, social participation, enforcement of self-management and personal recovery, and physical health (van Erp & Couwenberg, n.d.). Owing to the chronic nature of SMI, the individual demand for care, the severity and complexity of the problem, and the effects of the illness on the person's day-to-day life, the PMHNPs in the Netherlands regularly fulfill the role of coordinating practitioner and provide the main part of the treatment. This role fits in their area of expertise (Kappert & de Hoop, 2019). Besides PMHNPs, psychiatrists and health care psychologists are the most common practitioners fulfilling a role in treatment.

How patients perceive the value of the PMHNP is hardly known. No literature exists around the perception of the PMHNP care in the Netherlands. Wand et al. (2016) referring to care in Australia did incorporate the patient's perspective by evaluating a NP-led emergency department mental health care. Most of the patients valued the patience, time taken to listen and exploration of their situation by the mental health liaison nurse. Participants felt understood and reassured, and they considered the therapeutic communication skills of this professional as beneficial.

Regarding the PMHNP providing care for people suffering with SMI, only one study (also referring to care in Australia) has explored the patients' perspective on the value of two PMHNP candidate practices in the domain of physical health care outcomes in community mental health service (Furness et al., 2020).

None of the above-mentioned studies give in-depth insight into the experiences, values, and meanings of people with SMI with respect to the care provided by PMHNPs. Giving voice to patients' lived experiences will support further understanding of the PMHNPs’ added value to patient care.

## Method

### Aim

This study aimed to explore what meaning people with SMI associate with their experiences with the treatment and support provided by a PMHNP.

### Design

We used a qualitative design from a phenomenological and interpretative research paradigm because this perspective enables to explore interpretations and meanings of individuals' lived experiences (Polit & Beck, 2017, p. 465–472). To enable participants to recount their experiences as fully as possible, in-depth interviews, specifically individual interviews followed by focus group interviews, formed the main data source. To enhance understanding of the experiences, participants were invited to explicate nonverbally—using metaphors and pictures—what was in their “mind's eye.” Symbolism provides means of sharing one's experiences in a nonverbal way (Edward & Welch, 2011).

This manuscript adheres to the consolidated criteria for reporting qualitative research guidelines (Tong et al., 2007).

### Sampling and selection procedure

A purposive sample of people with SMI who had received inpatient or outpatient care by a PMHNP as a coordinating practitioner for at least 1 year was recruited from in total six mental health care organizations geographically spread across the Netherlands. Of these six, three organizations recruited participants for the individual interviews, and three other organizations recruited participants for the focus group interviews.PMHNPs of the six organizations acted as recruiters. They selected and invited candidate participants who met the criteria and gave them an information sheet containing details of the study.

Dutch-speaking adults of all ages who were expected to be able to critically reflect on their experiences were eligible for the interviews. Regarding the focus group interviews, candidates had to be able to listen and talk in a group environment. Those who wished to participate provided consent for passing on contact details by their PMHNP, after which the principal researcher contacted them by telephone to provide further information and answer questions. Participants signed an informed consent form before the start of the interview.

### Data collection

Data were consecutively collected by individual interviews conducted between March and October 2021, followed by focus group interviews in the first quarter of 2022.

#### Individual interviews

**Figure 1.**
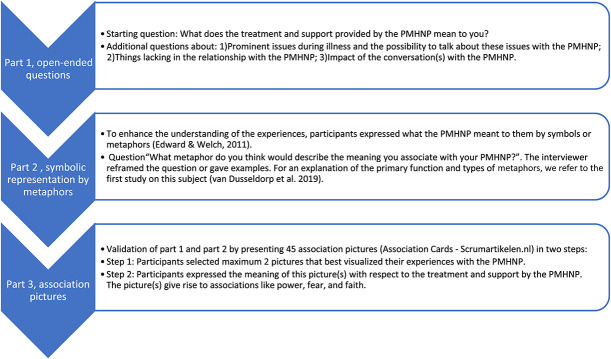
Flow diagram of the three parts of the in-depth individual interviews.

The individual interviews were guided by the same interview guide as used in previous studies (van Dusseldorp et al., 2019, 2020). A mental health expert checked whether this interview guide corresponded as closely as possible with this study's population, while the principal researcher explored specific literature regarding needs and problems of people with SMI (Delespaul & de Consensusgroep, 2013; van Erp & Couwenberg, n.d.). This did not lead to adaptation of the guide. Each individual interview consisted of three parts: (1) open-ended questions, starting question “What does the treatment and support provided by the PMHNP mean to you?” (2) symbolic representations by metaphors, starting question “What metaphor do you think would describe the meaning you associate with your PMHNP?” and (3) association pictures, questions “Please, select the picture that best visualizes your experience with the PMHNP” and “What is the meaning of this picture with respect to the treatment by your PMHNP?” (Figure 1).

Each participant was interviewed at home. The interviews lasted approximately 60 min and were audio-recorded.

#### Focus group interviews

The focus group interviews aimed to expand the exploration of the meanings of people with SMI and explore to what extent the results of the individual interviews could be confirmed. Based on broad experience with this method, the principal researcher and a senior researcher developed an adjusted interview guide that allowed for everybody to take part equally and was attuned to the aim, limited duration of the meeting, and the possible level of group interaction. Therefore, each focus group session consisted of two parts: (1) Participants introduced themselves and told briefly what the PMHNP meant to them and (2) exchanging of experiences with the treatment and support by the PMHNP based on the personally selected “association picture.” The sessions were mediated by the senior researcher and seconded by the principal researcher.

Focus group sessions were held at locations of the participating mental health organizations, lasted on average 60 min, and were audio-recorded.

### Research team

The research team consisted of a principal researcher, a research assistant, a senior researcher, and a research steering board. The principal researcher is a nurse (np) and a nurse scientist. The senior researcher is a nurse (np), too, and professor of person-centered (palliative) care at a University of Applied Science. During the study, both were employed by the affiliated University Medical Centre, Expertise Centre for Pain and Palliative Medicine. The research assistant is a senior lecturer in research techniques at the Master Advanced Nursing Practice. The steering board consisted of a Program Director Master Advanced Nursing Practice; a PMHNP who is also a senior researcher psychiatric mental health care and visiting professor at a University in Belgium; and an associate professor of Skill Mix Change.

Both researchers are women and experienced in research and in interviewing vulnerable people. They had no relationship with the participants before the study commencement. At start of the interviews, participants were informed about the background and occupation of the researchers.

### Ethical considerations

The affiliated University Medical Centre's Medical Research Ethics Committee (MCMREC) concluded that this study did not fall within the scope of the Dutch Medical Research Involving Human Subjects Act (Wet Medischwetenschappelijk Onderzoek met mensen). Therefore, further screening of the study protocol was not required (file number 2020-7019). In addition, as suggested by the MCMREC, the study protocol was approved by the research committees of the six participating organizations. To protect the participants' identities, ID numbers were used throughout the interview transcriptions. Participation was voluntary; participants could withdraw from the study at any time and without giving any reason. This decision would not affect their care in any way, as described (in the Netherlands) as an obligatory part of the informed consent.

### Data analysis

Data were analyzed using the identical method as in previous studies (van Dusseldorp et al., 2019, 2020), i.e., Colaizzi's seven-step method (Polit & Beck, 2017, p. 540), and the MIP (Edward & Welch, 2011; Tables 1 and 2). Participants of the individual interviews are indicated as individual female (IF) or individual male (IM) and participants of the focus group interviews as focus female (FF) or focus male.

**Table 1.** Colaizzi's seven-step method with inclusion of an additional step (Edward & Welch, 2011; Polit & Beck, 2017)

Colaizzi's Seven-Step Method of Phenomenological Enquiry With the Inclusion of an Additional Step
1. Transcribing all the subjects' descriptions
2. Extracting significant statements, i.e., statements that directly relate to the phenomenon under investigation
3. Formulating meanings for each significant statement
4. Organizing formulated meanings into clusters or themes
5. Integrating results into exhaustive description of the phenomenon
6. Additional step—interpretative analysis of symbolic representations
7. Identifying the fundamental structure of the phenomenon
8. Returning to the participants for validation of the findings

**Table 2.** Four steps of the MIP (Pragglejaz Group, 2007)

Four Steps of the MIP
1. Read the entire text discourse to establish a general understanding of the meaning
2. Determine the lexical units in the text discourse
3. (a) For each lexical unit in the text, establish its meaning in the context, that is, how it applies to an entity, relation, or attribute in the situation evoked by the text (contextual meaning. (b) For each lexical unit, determine if it has a more basic contemporary meaning in other contexts than the one in the given context. For our purposes, basic meanings tend to be more concrete (what they evoke is easier to imagine, see, hear, smell, and taste); related to bodily action; more precise (as opposed to vague); and historically older. (c) If the lexical unit has a more basic current contemporary meaning in other contexts than the given context, decide whether the contextual meaning contrasts with the basic meaning but can be understood in comparison with it.
4. If yes, mark the lexical unit as metaphorical

Note: MIP = metaphor identification procedure.

Computer software Atlas.ti version 9.1.6. was used to manage and analyze the data.

### Rigor

To assure trustworthiness of the data collection and analysis, the principal researcher used the following procedures: deidentifying the transcription of each interview, thick description, bracketing by maintaining a reflexive journal, making field notes after each interview, peer review and debriefing with MG, and a member check (Polit & Beck, 2017). During the data collection period, the principal researcher and research assistant independently applied steps 1–3 of Colaizzi's method to already obtained data. The senior researcher reviewed the analysis of the first four individual interviews and discussed with the principal researcher and research assistant the analysis of two randomly chosen other interviews. Simultaneously with steps 2 and 3, principal researcher and research assistant separately coded metaphorical expressions as “metaphor,” after which they compared and discussed the statements and determined the meanings. Disagreements were resolved through discussion. Next, steps 4, 5, and 7 of Colaizzi's method were performed through collaboration between principal researcher, research assistant, and senior researcher. The MIP (step 6) was initially performed by the principal researcher. Through discussion with the senior researcher, consensus was reached.

Data saturation of the individual interviews was reached after 16 fully transcribed interviews had been analyzed. In addition, two more interviews were held.

Finally, by way of a member check (step 8), the findings of the 18 individual interviews were presented to the interviewees in a table containing emerged meanings, themes, and fundamental themes (Edward & Welch, 2011; Polit & Beck, 2017). By means of a reply form, the participants could comment on the researchers' interpretations. Six participants responded to the member check. All but one “totally agreed” or “agreed” with the description of the (fundamental) themes. The responses did not lead to adaptation of the results. In addition, analysis of the focus group interviews in general confirmed the findings of the individual interviews.

## Results

### Characteristics of the participants

In total, 32 persons were interviewed; 18 individually and 14 different persons in the three focus group interviews. The distributions of the interviewees across the PMHNPs were as follows: Nine PMHNPs recruited the participants for the individual interviews, and eight PMHNPs recruited the focus group participants. During the interviews, demographic characteristics were collected. However, because not everyone was willing to share marital status and diagnosis, we can only describe characteristics collected at first hand. The mean age of the participants was 48 years (range 24–71), and 17 (53.1%) were men. Most of them (*n* = 21) lived independently, received ambulatory care, or received outpatient care. One participant lived in a professionally supported and secure small-scale housing project and received ambulatory care; 10 lived in a mental health care institution. Diagnoses shared with the interviewer varied from depressive disorder, eating disorder, post traumatic stress disorder, bipolar disorder, anxiety disorder, to paranoid schizophrenia. Duration of contact with the PMHNP varied from 1 to 8 years.

### Fundamental themes

Analysis of the interviews revealed eight fundamental themes, composed of 24 theme clusters.

1. Impact of the PMHNP on well-being.
2. Feeling connected with the PMHNP.
3. Feeling acknowledged by the PMHNP.
4. The PMHNP's care needed.
5. Perception of the PMHNP as a person.
6. Shared decision-making.
7. PMHNP's expertise.
8. Flexibility of contact with the PMHNP.

See Table 3 for a thematic map of these themes and theme clusters.

**Table 3.** Thematic map of emerged fundamental themes and theme clusters

Fundamental Themes	Theme Clusters	Examples of Formulated Meanings
Impact PMHNP on well-being	Acceptation	PMHNP helps with acceptance of self
	Pushing boundaries	PMHNP encourages pushing boundaries
	Empowered	PMHNP increases self-confidence and stability
	Changing perspective	PMHNP helps put things into perspective, normalize and see things differently
Feeling connected with the PMHNP	Trust	Having confidence in the PMHNP
	Humanity–equality	Experiencing equality
	Involvement	PMHNP shows commitment and genuine interest
	Click with the PMHNP	Feeling click with PMHNP, having nice contact
Feeling acknowledged by the PMHNP	Be human	Being able to be yourself, being seen as a full person
	Being taken seriously	PMHNP shows consideration for the situation and needs
PMHNP's care needed	Role in the treatment	Collaboration focuses on meaning, the positive, what is achievable and desirable
	Other dialog partners	In addition to PMHNP, support from GP, peers, or psychologist
	Avoid discussing with the PMHNP	Medication use is not always discussed with the PMHNP
Perception of the PMHNP as a person	Perception of the working alliance	PMHNP does not judge, helps search for solutions
	PMHNP as a person	PMHNP is patient, open, driven, and practical
	Shortcomings of the PMHNP	PMHNP is sometimes forgetful or arrives late
Shared decision-making	Decision support	PMHNP gives direction, helps to make choices, leaves control with the patient as much as possible
	Discussing	Listen to each other, consult, take each other's opinions seriously, look for solutions together
PMHNP's expertise	General expertise	PMHNP combines medical and psychological knowledge, gives advice and information
	Communication	PMHNP can explain things well, sees through matters, and discusses them in a pleasant manner
	Medication	PMHNP knows a lot about the (side) effects of medication, looks critically at use, and can prescribe medication
Flexibility of contact with the PMHNP	Need for contact	PMHNP can be reached in between Appointments are by mutual agreement
	Frequency of the contact	Contact needed with emotional or acute issues
	Duration	Length of appointment depends on the subject

Note: GP = general practitioner; PMHNP = psychiatric mental health nurse practitioner.

#### Impact on well-being

Some participants said that through contact with the PMHNP they could better understand the disease, cope with, and manage the consequences for daily life activities. “*She teaches me to deal with myself, that's very nice*.” (IF). Moreover, connection with the PMHNP was said to enhance self-confidence and self-assurance, which in turn contributes to self-acceptance.

As for pushing boundaries, the PMHNP stimulates and challenges patients to take steps forward by showing possibilities or opening new perspectives in dealing with vulnerabilities.

If I start telling my story to my PMHNP and she stimulates me at certain points, then lights go on in me and I try to do things differently next time. For example, I stand up for myself and start to feel better. (IF)

Many participants experienced the PMHNP's care as a great help and a source of empowerment. This created confidence, emotional stability, and support in expressing emotions.

Guidance of the PMHNP gave them self-management skills and contributed to the recovery process. Expressions such as “*She says ‘what you are doing now is really ‘eating disturbed’ and you would be better off shaping this and that*’, *then I dare do that more easily*.” (IF) and “*He helps me to find strength again to just keep going*.” (IM) illustrate this sentiment.

As for changing perspective, almost all participants found the PMHNP's alternative interpretations of reality clarifying. The PMHNP giving examples of his or her personal life positively influenced the way they assessed themselves and their situation: “… *For I am very much inclined to think ‘Oh, it’s all my sick mind’*. *But she can normalize things very much by saying ‘yes, that happens in my head as well’. For instance, this week we talked about choice stress in the supermarket. I told her I'm going crazy because I see 25 kinds of peanut butter. But then she says, ‘do you really think you are the only one just because you have autism? That happens to me as well’.*” (IF)

#### Feeling connected

Trust in the PMHNP was said to be important for feeling at ease and sharing problems and emotions openly with the PMHNP. “*She is actually my confidant when I have issues with food. You just don't share this so easily with people who don't understand eating problems*.” (IF) Many participants experienced trust right from the start of contact with the PMHNP, whereas for others, their trust had to grow, and one could be reluctant at first to discuss private matters. Good communication and PMHNP's expertise helped to strengthen mutual confidence.

Most of the participants highly valued the humanity and equality in the working alliance with the PMHNP. As one of them put it: *“… that's also a bit of equality, when we just talk about house, garden and kitchen things, and not necessarily about things I need help with. That's nice, because you feel more like a normal person instead of a patient*.” (IF)

Another aspect most participants appreciated is the PMHNP's personal involvement and sincere interest in one's personal life and feelings. The meaning of this experience is illustrated by expressions such as “*She said ‘whatever you choose, I'm behind you and we're going with you’*.” (IF) and “She always reaches out to me when I need it and also when I don't need it or don't think I need it.” (IF) and “*… when I tell her ‘I’*ve been in contact with the end-of-live clinic’, then she said ‘I so hope you live’. I thought yes, that’s what I really want to hear.” (IF).

Several participants “clicked” right away with the PMHNP, had a good relationship, and shared an affinity. This enhanced their feelings of trust, openness, and commitment.

… working on your goals … It’s nicer if there really is a click, it goes a little more automatically. (IF)

#### Feeling acknowledged

Participants found it highly important to be treated as a full-fledged person, not as “a patient who is ill.” Experiencing that “nothing is wrong with me, just the way you are,” enhances the feeling of being human and having an identity and dignity. Sharing personal situations with the PMHNP contributes to this perception.

… He says ‘hey, we also make lists at home, and my son this and my daughter that’. Then I think, yes, there are more people who solve it the same way. I just really enjoy that. (IF)

Concerning being taken seriously, several participants valued the consideration, compassion, and acknowledgment by the PMHNP for their situation and needs.

I really needed someone that I trusted and could talk to. Just say everything. I tried to trust him because of a radiance, a calm that looked seriously into my problems. (IM)

#### Care needed

The PMHNP's role in the treatment depends on one's needs, problems, struggles, and situation. The PMHNP provides therapy, tools, and advice and monitors mood or medication usage. Many participants highly appreciated the focus on the positive and the possible, which contributed to a meaningful and worthwhile life.

It is very nice to notice that she looks with me at what is important to me for my autism instead of what the protocol says. She really looks at what I need and yes that is very nice. (IF)

Some of the interviewees had decided to speak about their problems regarding social relations, religion, or daily life with someone else than the PMHNP, such as the general practitioner, fellow patients, or a vicar. In some cases, participants also received treatment and support from a psychologist or a community psychiatric mental health nurse.

A few participants said not to speak openly (yet) with their PMHNP about intimate issues or medication usage, such as overdosing or stop taking medication of one's own will. They explained this behavior because of the fear this may lead to the decision of the NP to order a clinical admission.

#### Perception of the PMHNP as a person

All participants positively valued feeling being understood and not being judged by the PMHNP. The PMHNP encouraged them to talk about their feelings and finding solutions together. One participant described the working alliance as a “we” feeling, like several other participants: “*She thinks along with me, and asks ‘what can we do to change this or what do you think we can do*?’” (IF)

The PMHNP's humanity meant a great deal to the interviewees. His or her personal characteristics, such as being patient, friendly, having a practical attitude, being motivated, reliable, open, and transparent, were found important to the treatment and support.

Minor shortcomings mentioned were being forgetful regarding prescriptions or agreements about weight gain. Whereas most interviewees found nothing lacking in the relationship with the PMHNP, others described situations in which they felt misunderstood about their religion, felt a lack of personal attention, or even felt a lack of confidence because in one's own view the PMHNP did not perform the job properly.

#### Shared decision-making

The meaning participants associate with decision support focused on the PMHNP providing direction and helping to make one's own choices. “*Someone who thinks along, not someone who offers ready-made solutions*.” (IM) Staying in control as much as possible was referred to as well. “*I wanted to change the dosage of the medication, but was also scared of the possible consequences. He [the PMHNP] said to me ‘It’s okay to decrease the dose but think carefully about it’. He leaves the final decision with me. If I don't want it, it won’t happen*.” (IF) Furthermore, participants appreciated that the PMHNP focuses on one's needs and wishes and that one can set the pace at which things happen. As someone said: “*I can indicate where my interests lie at that moment in time. And that we will investigate, that part*.” (FF).

Being in dialog—that is, listening respectfully to each other, taking each other's arguments seriously, and sometimes disagreeing with each other—was found important. Looking for solutions together stimulates one in a positive way:

If she suggests to take an extra snack at a certain moment, but I don’t dare to do so, I just tell her that and we see why that is and if it is possible to take something else. (IF)

#### Expertise

Participants valued the PMHNP's expertise about their disease, combining medical and psychiatric knowledge, approaching problems from different perspectives, giving advice, and psychoeducation. One participant said: “*It’s just nice that he can look at me from a certain medical background, so to speak. In that way he can understand certain things about me that perhaps people do not understand who do not have that background. And also thinks along with me about improvements that I can make.*” (IM)

The PMHNP's communication skills were described as assisting one in clarifying thoughts. As someone put it: “*She can say just the right things, while I couldn't articulate it so well. … She sees through those things (issues with my mother) and she just knows how to address it well*.” (IF) Participants also appreciated the close monitoring of their well-being and discussing issues in a pleasant way.

The meanings interviewees associate with the PMHNP's expertise about medication concerned knowledge about effects and side effects, a critical review of medication intake (dose and reduction), and the authority for prescribing medication. Talking with the PMHNP about medication use was appreciated, e.g., in case of sleeping problems or side effects. Openly discussing with the PMHNP the pros and cons of medication was found helpful. As one participant put it*:* “*I like that you can always discuss medication and also lab or something like that. They do know side effects and that sort of thing.*” (IF)

#### Flexibility of contact

Participants appreciated the PMHNP being accessible between appointments, by means of mail, app, or telephone for (urgent) questions or problems. They mentioned that the PMHNP always responded as quickly as possible.

… *She knows you very well and she knows that when I contact her it is really necessary to call me back, text me back. She always takes it seriously*. (IF) Clinically admitted patients could just drop in at the PMHNP’s office for a ‘small talk’.

Some participants said to highly value the PMHNP's expertise and attention in case of suicidal thoughts, emotional issues, or feeling depressed: “*I can contact him if necessary. That's important*.” (IF). Others mentioned they can discuss some things only or at first with the PMHNP: “*Some things I can only discuss with her, because she has known me for so long, and knows me so well*.” (IF)

The frequency of contact with the PMHNP differed. For some, it is regularly every month, while others schedule the next appointment themselves, depending on their needs. The duration of appointments can vary just as the frequency, depending on what is going on.

### Metaphors

The MIP analysis revealed six metaphors: PMHNP is a travel aid, means trust, is a combat unit, means hope, is an exhaust valve, and is a helpdesk/encyclopedia. Each of the six metaphors is described below.

#### Travel aid

In this metaphor, the PMHNP was depersonalized into a lighthouse, safe haven, a rock in the surf, and anchor. When one feels upset, confused, or lost, contact with the PMHNP makes one feel protected and stable: “He is an anchor; at the moment I threaten to float away then the anchor comes, ‘*think what you're doing huh?’. And yes I'm getting a little bit closer. … He'll let me have my way but not drift too far.*” (IF)

#### Trust

One participant described the PMHNP's care as “a warm coat.” The PMHNP makes one feel safe and gives a secured, enclosed feeling.

#### Combat unit

One participant positively visualized the collaboration with the PMHNP as acting like a common front resisting the illness. On the other hand, another participant was quite negative: “*the PMHNP is a demon who wants to destroy me.*” (IM)

#### Hope

Some participants saw the PMHNP as a light in the dark. In dark, difficult situations one can reach out to the PMHNP who gives directions and hope. One person described it this way: “*When I was depressed and thought ‘death is the only option’, at that moment she stood next to me and gave me hope. Together with her I'm rediscovering life.*” (IF)

#### Exhaust valve

A few participants gave the PMHNP a more technical quality by referring to a *sounding board*, *outlet*, or *a sympathetic ear*. Another participant associated the PMHNP with *a hot air balloon.* All these meanings value the PMHNP as a sparring partner and the contact as a means to let of steam, while the PMHNP listens attentively to what is on your mind.

… Without saying how things should be different or better. However, when I am bothered with something and ask myself ‘how to move forward?’ … Not that he has a ready-made solution, but that he thinks along with me. (IM)

#### Helpdesk/encyclopedia

This metaphor of knowledge (transfer) signified another aspect of the PMHNP as experienced by one participant. For this person, the PMHNP is a centipede, someone with expertise and knowledge from different angles, who pushes and stimulates to get life back on track.

### Symbolic representation by association pictures

**Figure 2.**
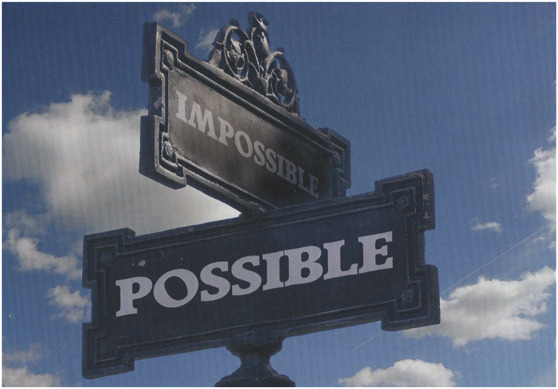
“Looking at the possibilities together, the PMHNP makes the impossible possible.”

Of the 45 association pictures presented to the participants, the most selected one (6×) represented the contact with the PMHNP as “*looking at the possibilities together, the PMHNP makes the impossible possible*” (Figure 2). Many other different pictures were selected by one or two persons; yet, these all had a similar underlying meaning or intention: namely the PMHNP providing support and guidance in, among other things, making choices, showing emotions, opening “the cage,” finding new tools, giving directions, and a runway to solve the puzzle of life.

## Discussion

The findings of this explorative study about patients' perceptions of the PMHNP care suggest that people with SMI highly appreciate the PMHNP for eight fundamental themes: (1) impact on well-being, (2) feeling connected, (3) feeling acknowledged, (4) care needed, (5) perception of the PMHNP as a person, (6) shared decision-making, (7) expertise, and (8) flexibility of contact.

In general, the meanings patients in this study associate with the treatment and support provided by a PMHNP show similarities with those found in previous qualitative studies by van Dusseldorp et al. (2019, 2020). Although the populations differed—life-threatening illness, chronic illness and SMI—the core meanings seemed the same.

Caring for people with SMI demands specific skills. Many of them suffer from the negative impact of internalized or self-stigma; they believe in or apply negative public stereotypes, prejudice, and discrimination to themselves. Various recovery-orientated care programs have shown that aiming for the positive side is an imported intervention target both for mental health care professionals and their patients (Catalano et al., 2021; Cunningham & Lucksted, 2017; Stacy & Rosenheck, 2019). According to Stacy and Rosenheck (2019), the focus of recovery-promoting efforts lies on fostering hope, strengths, empowerment, and self-direction. The participants in our study indeed confirmed this by telling that the PMHNP's support made them feel more empowered, confident, human, and equal. The PMHNP’s decision support, interest in one's personal life, and—perhaps the most important aspect—the normalizing of situations seem to strengthen the recovery-promoting efforts. In line with Cunningham and Lucksted (2017), who addressed the importance of fostering flexibility to help reduce internalized stigma, participants in our study appreciated the PMHNP who stimulates changing perspectives and pushing boundaries. The above-mentioned considerations may suggest that the PMHNP incorporates these techniques or strategies quite naturally within the working alliance with people with SMI.

## Limitations

Some methodological limitations should be considered while interpreting the results. Although additional quantitative data might strengthen the emerged evidence, we trust that our findings can be transferred to all PMHNPs providing care to people with SMI in the Netherlands. For one thing, the participants were recruited from six mental health care organizations by their own PMHNPs, taking into account the inclusion criteria formulated in the study protocol. Still, they needed to assess whether a potential participant was able to reflect on personal experiences and had the psychological and social strength to take part in an individual or group interview. This may have led to selection bias, either in a positive direction or in an unknown direction. To minimize the risk of bias, the principal researcher had encouraged PMHNPs to recruit who dared to be critical. Despite these potential limitations, we have faith in the trustworthiness of the study. The rigor of the data collection and data analysis and the applied steps and methodologies strengthen the value of the achieved insight.

Our findings represent the situation in the Netherlands, in which the PMHNPs act on the intersection of cure and care, blending medical, psychiatric, and nursing competencies and fulfill the role of coordinating practitioner. Their tasks and roles cannot be completely generalized to a wider international context.

## Conclusion

To the best of our knowledge, this is the first study exploring in depth what meaning people with SMI associate with the treatment and support by a PMHNP. Although the perspective of one participant ran counter to that of the whole group, we may conclude that the PMHNP's treatment and support is highly valued for the impact on patient's well-being. One feels empowered, human, understood, and challenged thanks to the connection with and recognition from the PMHNP. Despite some slight methodological limitations, this study adds to the body of knowledge on PMHNPs’ roles and practices.

## Implications for practice

People with SMI highly appreciate the fact that the PMHNP normalizes situations to help reduce internalized stigma. Furthermore, being taken seriously and the PMHNP being interested in one's personal live were appreciated. We recommend, therefore, that PMHNPs reflect on these appreciated issues and that the curricula of Advanced Nursing Practice programs pay more attention to these issues. Patients highly value the emotional aspects in the working alliance with their PMHNP. Therefore these aspects need to be addressed in various situations, such as policy making, education, PMHNPs' daily practice and collaboration with other professionals. Participants' views in the complete set of three studies were focused on the NP. Future research should therefore explore patients' views on the care provided by other professionals in similar or different settings.
